# Human parvovirus B19 and pediatric rheumatologic disease

**DOI:** 10.1186/1546-0096-10-S1-A102

**Published:** 2012-07-13

**Authors:** Trevor Davis, Caitlin Sgarlat, Jorge Lopez, Marc Natter, Laurie Miller

**Affiliations:** 1 Tufts, Boston, MA, USA

## Purpose

Human Parvovirus B19 causes a self-limited febrile illness with a characteristic exanthem and occasional acute arthritis. B19 infection has also been associated with multiple rheumatic diseases. Reports indicate that in patients with positive B19 PCR up to ~1/3 have negative IgM and positive IgG to B19. However little is known about how these findings might relate to rheumatologic symptoms, especially in children.

## Methods

The records of 9 patients presenting to a pediatric rheumatology clinic with acute onset arthralgia or arthritis and positive B19 PCR from 2007-9 were examined retrospectively. Demographic information, clinical course, physical exam, and laboratory data including B19 serology were recorded. The IgM-positive subgroup was compared to the IgM-negative subgroup for differences in any of the other variables by the two sided Fisher exact test or independent t-test.

## Results

The 9 children’s (3M:6F) ages ranged from 7-19 years. Presenting signs included arthralgia (100%), polyarthritis (67%) and enthesitis (22%). Presenting laboratory findings included elevated ESR and CRP (both 78%), positive ANA (33%), positive dsDNA (11%), RF (22%), APL antibodies (22%), and low C4 (11%). Six infected patients (66%) otherwise met classification criteria for systemic JIA (2), enthesitis-related arthritis (2), RF negative polyarticular JIA (1), or SLE (1). Patients’ B19 serology included 8 (89%) with positive IgG of which 4 (44%) had positive IgM. Five patients had negative IgM [1 each with SLE, enthisitis related arthritis (ERA), polyarticular JIA, and 2 systemic JIA]. Compared to patients with positive B19 IgM, those with negative IgM had higher ESR at presentation (mean+SD 82.4 + 53 vs. 10.7+9.3, p=0.04), trend to higher CRP values (91.4+ 85.1 vs. 5.7+8.5, p=0.09), and were more likely to be treated with steroids (p=0.04). In 6 patients (67%) the positive B19 PCR was not found to persist past 1 month and other clinical and laboratory findings resolved in <6 months. In the remaining 3 (1 each with polyarticular JIA, SLE, and ERA), the illness persisted past 6 months. The patient with SLE continues with a positive ANA years after presentation, although B19 PCR resolved in 6 months and both APL and dsDNA antibodies resolved within 1 year. In the patient with polyarticular JIA B19 PCR was still positive 3 months after presentation. Although her symptoms resolved quickly her inflammatory markers continued to be elevated. The patient with ERA is still being followed for his illness, now >6 months into disease. He still has enthesitis and is B27 positive but all other labwork is normal.

## Conclusion

Evidence of active B19 infection may be found in children with symptoms that mimic a variety of pediatric rheumatologic diseases, including systemic and polyarticular JIA, ERA, and SLE. Despite this, without persistence of positive B19 PCR, symptoms resolve relatively rapidly. However, persistence of B19 viremia may correlate with prolonged symptomatology and more aggressive treatment. Furthermore children with positive B19 PCR and negative IgM antibodies may have increased risk of a more severe presentation. Therefore obtaining and following B19 PCR is essential in suspected cases.

## Disclosure

Trevor Davis: None; Caitlin Sgarlat: None; Jorge Lopez: None; Marc Natter: None; Laurie Miller: None.

